# Strengthening the occupational and social participation of multiple sclerosis patients - design of a multicenter, parallel-group randomized controlled trial (MSnetWork-study)

**DOI:** 10.1186/s12883-022-02947-0

**Published:** 2022-12-09

**Authors:** Sandra Meyer-Moock, Susan Raths, Katharina Strunk, Bernward Siebert, Katrin Hinkfoth, Markus Weih, Steffen Fleßa, Thomas Kohlmann

**Affiliations:** 1 Institute for Community Medicine, University Medicine of Greifswald, Greifswald, Germany; 2 Department of Health Care Management, University of Greifswald, Greifswald, Germany; 3 GWQ ServicePlus AG, Society for Efficiency and Quality in Health Insurance Funds, Düsseldorf, Germany; 4 VDBW - Professional Association of German Occupational Physicians, Berlin, Germany; 5 BDN - Professional Association of Neurologists Mecklenburg Western Pomerania, Ribnitz-Damgarten, Germany; 6 BDN/BVDN - Professional Association of Neurologists Bavaria, MVZ Medic Center Schöll&Kollegen, Nürnberg, Germany

**Keywords:** Multiple sclerosis, Health-related quality of life (HRQoL), Study protocol, Health economic research, Health service research, Germany, Incapacity to work, Ability to work

## Abstract

**Background:**

Multiple Sclerosis is an autoimmune inflammatory disease of the central nervous system that often leads to premature incapacity for work. Therefore, the MSnetWork project implements a new form of care and pursues the goal of maintaining or even improving the state of health of MS patients and having a positive influence on their ability to work as well as their participation in social life. A network of neurologists, occupational health and rehabilitation physicians, psychologists, and social insurance suppliers provide patients with targeted services that have not previously been part of standard care. According to the patient’s needs treatment options will be identified and initiated.

**Methods:**

The MSnetWork study is designed as a multicenter randomized controlled trial, with two parallel groups (randomization at the patient level with 1:1 allocation ratio, planned *N* = 950, duration of study participation 24 months). After 12 months, the patients in the control group will also receive the interventions. The primary outcome is the number of sick leave days. Secondary outcomes are health-related quality of life, physical, affective and cognitive status, fatigue, costs of incapacity to work, treatment costs, out-of-pocket costs, self-efficacy, and patient satisfaction with therapy.

Intervention effects are analyzed by a parallel-group comparison between the intervention and the control group. Furthermore, the long-term effects within the intervention group will be observed and a pre-post comparison of the control group, before and after receiving the intervention in MSnetWork, will be performed.

**Discussion:**

Due to the multiple approaches to patient-centered, multidisciplinary MS care, MSnetWork can be considered a complex intervention. The study design and linkage of comprehensive, patient-specific primary and secondary data in an outpatient setting enable the evaluation of this complex intervention, both on a qualitative and quantitative level. The basic assumption is a positive effect on the prevention or reduction of incapacity for work as well as on the patients’ quality of life. If the project proves to be a success, MSnetWork could be adapted for the treatment of other chronic diseases with an impact on the ability to work and quality of life.

**Trial registration:**

The trial MSnetWork has been retrospectively registered in the German Clinical Trials Register (DRKS) since 08.07.2022 with the ID DRKS00025451.

## Background

Multiple sclerosis (MS) is a chronic inflammatory autoimmune disease of the central nervous system that affects more than 223,000 statutorily insured people in Germany [1]. The disease is usually diagnosed between the ages of 20 and 40 and is associated with severe physical, cognitive, and social impairments [2]. Treatment options are defined in guidelines and focus on recovery from attacks, slowing down the disease progression and managing MS symptoms [3]; by now there is no known cure for MS.

For many MS patients, the disease leads to a significant reduction in their working ability. As a result, the proportion of patients with part-time jobs, disability pensions, or premature retirement increases in the course and severity of the MS [2]. Disease-specific factors such as mobility limitations, fatigue, and cognitive impairments are primarily responsible here. Comorbid depression, which is often accompanied with MS, also frequently leads to restrictions on the ability to work. However, working conditions also have an impact on employment prognosis, as in many cases there is a lack of understanding in physically demanding activities in the workplace and a lack of necessary flexibility to ensure the (continued) employment of people with MS [4]. This is precisely where the MSnetWork project comes in because it is less about the effect of individual measures or interventions on clinical disease parameters (e.g. relapse frequency), but rather about the effect on participation in private, social, and, in particular, vocational life and the associated long-term securing of the ability to work.

The MSnetWork project implements a new form of care and pursues the goal of maintaining or even improving the state of health of MS patients and having a positive influence on their ability to work as well as their participation in social life. A network of neurologists, occupational and rehabilitation physicians, psychologists, and social insurance suppliers provides patients with targeted services that have not previously been part of standard care and are directly tailored to the personal needs of the patients. According to the patient’s needs based on a systematic and regular assessment of functional capacity, the following treatment options will be identified, initiated, and coordinated by the neurologist:

- participation-oriented care by the MS-specialized neurologist,
- occupational health examination by an occupational physician,
- psycho-social and legal counseling from a qualified person,
- disease education by a neuropsychologist or a neurologist who is specialized in MS,
- MS-specific outpatient and inpatient rehabilitation by a rehabilitation physician.

Risks of loss of social participation as well as of inability to work will be identified and should be reduced by the interventions. The patient will be empowered to make informed decisions about their treatment options and will be supported in upholding the ability to work and to participate in social life.

The study was initiated by the Professional Association of German Neurologists (Berufsverband Deutscher Neurologen e.V., BDN) and is conducted together with GWQ ServicePlus AG as representative of a large number of statutory health insurance funds and the Professional Association of German Occupational Physicians (Verband Deutscher Betriebs- und Werkärzte e. V.). The University of Greifswald (UG) and the Institute for Community Medicine of the University Medicine of Greifswald (UMG) are responsible for the scientific and economic evaluation within MSnetWork.

## Methods / design

### Research questions and working hypotheses

The main aims of the study are (a) to improve or stabilize the health status of patients with MS and (b) to prevent or reduce their incapacity to work. Therefore, the intervention consists of a portfolio of measures to improve the coordination of specialist care as well as the early integration of socio-medical, occupational, psychosocial, rehabilitative, and health care services.

### Study design

**Fig. 1:**
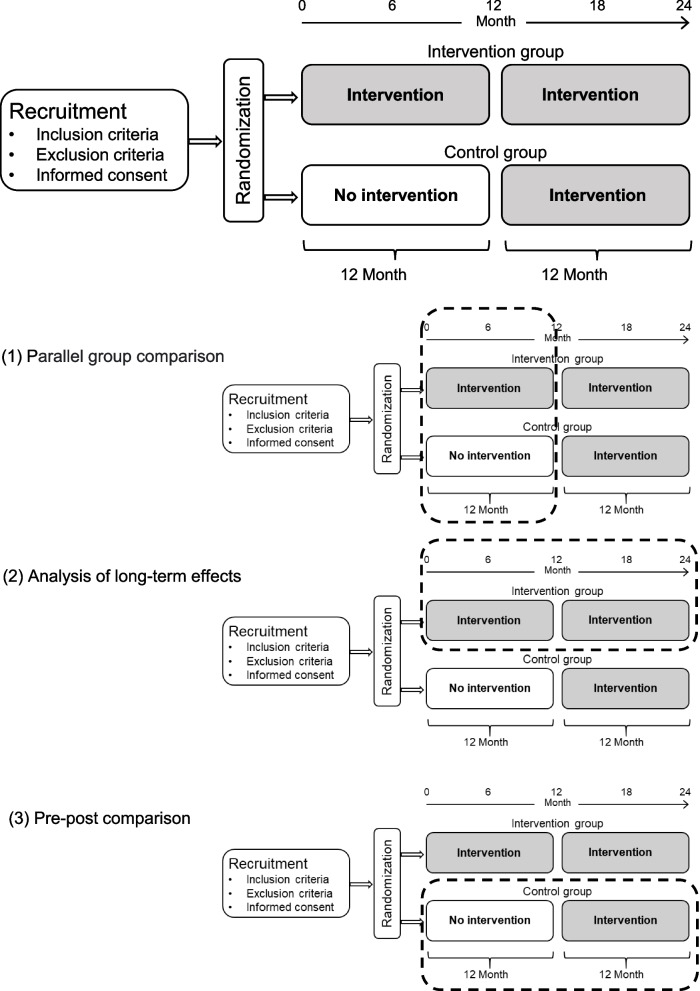
Interventions and timeline

The MSnetWork study is designed as a multicenter randomized controlled trial (RCT), with two parallel groups. The duration of study participation is 24 months. The patients are consecutively enrolled between January and December 2022, and assigned to an intervention group or a control group in a ratio of 1:1 after checking the inclusion and exclusion criteria. After 12 months, the patients in the control group will also receive the intervention measures, during the first 12 months they receive standard care. The study procedure planned is shown in Fig. 1.

The intervention effect is analyzed as follows:

1. Essentially, the intervention effect is carried out by analyzing the course of the primary and secondary outcomes in the intervention and control groups in the first 12 months (parallel-group comparison).
2. In the following 12 months, the intervention group continues to receive the intervention. Further analyses of the outcome variables in this time are particularly important to be able to check the stability of this effect over the time (long-term) to see if an effect of the intervention is present after the first 12 months.
3. The control group receives the intervention in the second half of the study period. The course of the primary and secondary outcomes in the control group between the first and second period (months 0 to 12 vs. months 13 to 24) will be analyzed either. Such a pre-post comparison is methodologically weaker than a parallel group comparison, but it offers the possibility to examine the effect of the intervention once again from an independent perspective.

### Subjects and eligibility criteria

Criteria for study inclusion comprise the following: (1) patients with a confirmed diagnosis of multiple sclerosis; (2) patients of working age (aged 18–65 years); (3) patients employed (part-time or full-time) or in study or vocational training.

Criteria for study exclusion comprise the following: (1) patients with MS who are no longer of working age (older than 65 years); (2) patients who are unable to work; (3) patients with severe impairment of independence (corresponds to care level 4 and 5 in German social law), and (4) patients with severe mental disorders.

### Sample size

The calculation of sample size was done for the analysis of the primary outcome “number of sick leave days” per patient within 12 months. The calculation assumes that the difference in sick leave days is to be an average of 5 days (or 20% of the average sick leave days for MS patients in Germany) between the intervention and the control group. This number is deduced from the German statistics on types of illness [5], with an annual average number of 25 days of incapacity to work due to MS among the statutorily insured persons. Further, based on a database analysis of the insurances taking part, up to 50% of the insured persons with MS do not have any days of incapacity to work in a 12-month observation period. Hence, the number of cases is calculated with a regression model for count data with “zero inflation”.

Depending on the assumption about the proportion of insured persons without sick leave days (30% vs. 50%), with a statistical power of 80%, and a 2-sided significance level of 0.05, the minimum sample size necessary to identify a group difference for the primary outcome of sick leave days is between 600 and 860. An expected drop-out of 20% in the course of the twelve-month comparison period leads to an increase in the net case numbers to the gross case numbers of *N* = 750 and *N* = 1075 (Table 1).

**Table 1:** Results of the calculations for determining sample size

Patients without days of incapacity to work within 12 months (in %)	Net number of cases	Gross number of cases^a^
30% 50%	600 860	750 1.075

^a^Drop out of 20% in 12 months

Therefore, we aim for an initial gross sample size of 950 cases in total (475 cases per group). This number of cases is somewhat closer to the upper limit and would thus make it possible to achieve the planned power even if the actual conditions deviate from the assumptions made.

The strategies to achieve adequate recruitment to reach the target sample size had been.

based on the number of patients being treated in the past in the neurological practices who would have fulfilled the eligibility criteria. Neurological practices will be identified with the help of the BDN, the Professional Association of Neurologists.

### Participant identification / recruitment strategy and randomization

Patients are recruited from neurological practices and outpatient clinics in Bavaria, Berlin, Brandenburg, Hesse, Baden-Württemberg, Schleswig-Holstein, North Rhine-Westphalia and Mecklenburg-Western Pomerania in Germany. Patients of a centre who fulfill the inclusion criteria and for whom there are no reasons for exclusion are consecutively included in the study. After information and obtaining consent to participate, patients are randomized into the intervention and control groups.

Concealed randomization with variable block length takes place at the patient level using an online tool implemented in the electronic health record system. The allocation sequence is generated by the UMG.

Patients can be enrolled via managed care contract with the participating health insurance companies or via a direct treatment contract with the physician (without the participation of a health insurance company).

### Study centres

The study centres take on essential functions in the area of patient recruitment (patient information, informed consent), the management and documentation of study-relevant procedures, data collection, and the forwarding of data, in addition to intervention-related services. Each practice receives detailed training with the study procedures in the context of virtual training sessions before the start of patient inclusion.

Physician practices or participating providers are reimbursed for enrolment and performance of individual intervention services on a per-service billing basis. The individual services provided per patient and the corresponding remuneration are presented transparently for the service providers via the patient platform used and invoiced to the project management. The preliminary calculation of compensation is primarily based on estimates of the time required for single services.

### Intervention

A MS-specialized neurologist examines the cognitive and physical status of the MS patient using standardized assessments like the Symbol Digit Modalities Test (SDMT) [6], Expanded Disability Status Scale (EDSS) [7], 9-Hole Peg Test (9-HPT), and Timed 25-Foot Walk (T25-FW) of the Multiple Sclerosis Functional Composite (MSFC) [8–10] every six months. Additionally, as part of a quarterly consultation on health, affective, and participation status, the neurologist examines health-associated participation and workability. The quarterly medical consultation by the neurologist includes participation-, deficit- and resource-oriented therapy planning as well as progress monitoring with regard to health-associated participation and the preservation of the working ability. In addition, it serves to ensure the stabilization of the current treatment success and the optimization or adaptation of the therapy.

As a result, the neurologist identifies risks that influence health status, workability status, family status, and social status due to the living environment. Together with the patient, the physician discusses possible treatment options to improve health and prevent workability as well as plans therapy. The neurologist initiates and coordinates the interventions with other health care providers (e.g. occupational physician, psychologist, rehabilitation physician, pension insurance, health insurance company) based on the patient’s needs. After the intervention has taken place, or if additional treatment needs have been identified by the service providers during the intervention, the neurologist and the corresponding service provider can hold a teleconsiliary discussion on the findings to ensure the success of the treatment and to discuss the therapy goal. If the neurologist identifies an acute risk of incapacity or reduction in earning capacity during his*her examination, he*she can initiate a case conference together with the other players involved to discuss possible treatment options for acutely avoiding incapacity and loss of participation. The patient can be included in the case conference to ensure treatment adherence.

The neurologist has different treatment options to improve health and workability. If the treating neurologist identifies:

- health risk from employment and risk for disease exacerbation, the neurologist initiates an *occupational health examination* by a qualified occupational physician. The focus of the occupational health examination is to identify health destabilizing influences and to reduce them by occupational medical interventions tailored to the patient’s needs.
- deficits in coping with the disease during the examination that contribute to worsening health: the neurologist can initiate *disease education*. The focus is on teaching environmental and behavioral strategies for better coping with MS. Patient education promotes active engagement with MS and activation of self-efficacy and knowledge building. Patient education is offered by MS-specialized neurologists or MS-specialized neuro-psychologists, depending on regional needs. The counseling neurologists and neuro-psychologists are to be qualified by a specialized training module.
- risk of or present restriction for participation due to MS or symptoms accompanying MS (affective, cognitive disorders, physical deficits, etc.), which cannot be eliminated, compensated, or minimized by the previous interventions, the neurologist initiates *an inpatient, day-care, or outpatient rehabilitation* in an MS-specialized rehabilitation centre, depending on the patient’s need.
- a negative influence of the social and family environment during his examination, the neurologist initiates individual *psychosocial and legal counseling*. The focus of the consultation is on the individual identification of challenges in the family and social environment, support in determining and enforcing legal claims, and support offers. The counseling, support, and close follow-up help to improve health and reduce or eliminate the burden of the disease. Depending on the regional situation, counseling is provided by the MS self-help organization or medical assistant within the neurological practice. The counselors are qualified by proven MS-specific knowledge as well as by a qualified degree as a social worker; the medical assistant will be qualified by an appropriate advanced training module.

### Control condition

In the first 12 month, participants in the control group will not receive trial-related healthcare interventions, but “treatment as usual”, i.e. the standard healthcare as currently applied in the MS standard treatment in Germany. This comparator was chosen because the research question is whether the new intervention affects outcomes like days of incapacity of work, quality of life, and cost-effectiveness and for this a comparison to “usual care” is warranted.

**Fig. 2:**
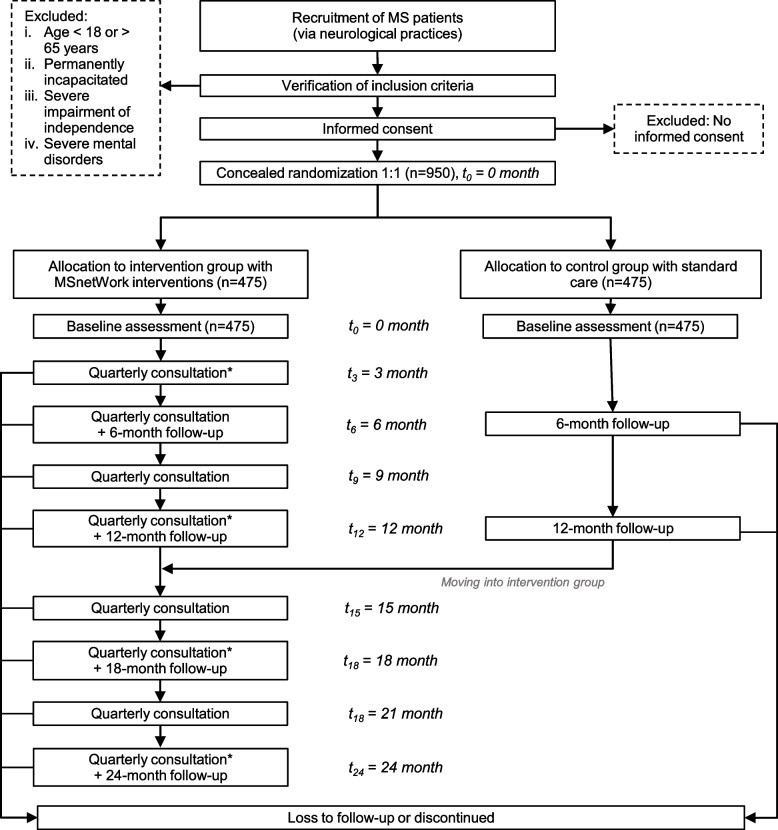
*Flow of participants in MSnetWork.* * The quarterly medical consultation by the neurologist includes participation-, deficit- and resource-oriented therapy planning and progress monitoring with regard to health-associated participation and the preservation of the working ability. In addition, it serves to ensure the stabilization of the current treatment success and the optimization or adaptation of the therapy

However, patients in the control group do also receive interventions after 12 months at the same frequency as participants in the intervention group (Fig. 2).

### Electronic health record

Networking and documentation, as well as data exchange between the players involved, take place by using an electronic health record. The online platform contains relevant data on the patient’s MS-associated health data and information and results on assessments for functional capacity as well as examinations from occupational physicians. In addition, the platform in which the patient’s record is embedded makes available group offers for disease education recognizable for the coordinating medical assistant of the neurological practice to enable rapid scheduling and therapy assignment. The data exchange is based on the already existing and legally provided structures of the patient’s electronic health record, is used only after the patient’s consent, is browser-based, and has the necessary interfaces for demand-oriented care.

### Outcomes

#### Primary outcome

The primary outcome is the number of MS-associated sick leave days at 12- and 24-month follow-ups. These data will be provided by the routine data of the health insurance funds. In addition, the days of incapacity to work will be collected with a baseline and follow-up patient questionnaire every six months, as well as via the digital patient file during the quarterly consultations (intervention group) or the functional assessments (control group). Self-reported primary data can be used for the analysis of patients with and without secondary data from the health insurance funds (inclusion via managed care contract or treatment contract), but are somewhat more susceptible to information bias in direct comparison (e.g. self-reporting bias, recall bias) [11].

#### Secondary outcomes

The secondary outcomes include primary data collected within the evaluation study and secondary data provided by the health insurance funds. For patients, primary data will be collected by a questionnaire at baseline and on 6-, 12-, 18-, and 24-month follow-up, that consist of the following:

1. Health-related quality of life is measured by the German version of the Multiple Sclerosis Quality of Life (MSQoL)-54 instrument [12], a diagnosis-specific MS questionnaire. It contains 52 items distributed into 12 scales, and two single items. In addition to the generic quality of life items retrieved from the SF-36, the questionnaire captures health aspects that are particularly significant for MS patients (18 items in the areas of health distress, sexual function, satisfaction with sexual function, overall quality of life, cognitive function, energy, pain, and social function).
2. The EQ-5D-5L is a preference-based HRQoL measure and is used to evaluate the generic quality of life. The EQ-5D-5L contains one item for each of the five dimensions mobility, self-care, usual activities, pain/discomfort, and anxiety/depression. The answers given to EQ-5D-5L can be converted into a utility score, the EQ-5D index. Additionally, the EQ-5D-5L includes a Visual Analog Scale (VAS), ranging from 0 (the worst possible health status) to 100 (the best possible health status) [13]
3. Affective status is evaluated by the short form of the Depression, Anxiety and Stress Scale (DASS) [14, 15]. The DASS comprises three subscales each consisting of 7 items: depression, anxiety, and stress.
4. Fatigue as a common symptom in MS patients [16] is measured with the Fatigue Scale for Motor and Cognitive Functions (FSMC] [17]. The 20-item scale contains cognitive and motor subscales with 10 items each.
5. A short scale measuring the subjective prognosis of gainful employment (SPE-scale) [18] is used to assess the likelihood of reduction of work capacity (3 Items).
6. Patients’ adherence to therapy and treatment is measured by the Adherence Assessment Questionnaire (AAQ) [19]. The AAQ consists of 11 items, including a visual analog scale.
7. Self-efficacy is carried out with the 13-item short form of the Coping Self-Efficacy Scale (CSES) [20]. The CSES measures one’s confidence in performing coping effectively with life challenges e.g. due to chronic illness and it can assess changes in coping self-efficacy (CSE) over time in intervention research.
8. The level of patient satisfaction with the new intervention is measured with an adapted version of the Patient Satisfaction Questionnaire (ZUF-8) [21, 22] The original version was adapted for use in outpatient care.
9. Out-of-pocket payments by households due to the MS disease are measured with 16 items. These include co-payments, monetary transport costs, and time losses. The items are based on the Multiple Sclerosis Health Resource Utilization Survey [23] but were adapted for the specific research design in MSnetWork.

Additionally, primary data collected as part of the intervention at neurological practices will also be used for the evaluation as secondary outcomes:

(10) The cognitive status is assessed by the Symbol Digit Modalities Test (SDMT) at baseline and on 6-, 12-, 18- and 24-month follow-up.
(11) The physical status is measured using the Expanded Disability Status Scale (EDSS), the 9-Hole Peg Test (9-HPT), and the Timed 25-Foot Walk (T25-FW) of the Multiple Sclerosis Functional Composite (MSFC) at baseline and on 6-, 12-, 18- and 24-month follow-up.

Secondary data of the patients are made available by the participating health insurance companies and consists of the following:

(12) Treatment costs and frequency: For each included patient, the data set contains the cost data on inpatient and outpatient care, and if applicable rehabilitation costs, medication, remedies (e.g. physiotherapy, occupational therapy), and aids as well as other payments. In addition, the GWQ Service Plus on behalf of the health insurance companies directly involved forms a match from their patient data of patients with MS that is as structurally identical as possible (propensity score matching by age, gender, 3-st. postcode, among other things), who are not included in the study. This quasi-experimental cohort study design allows further comparison between MS patients, with and without study participation, meaning between patients with and without receiving intervention services.
(13) Sick leave days and sickness allowance: The data set contains the sick leave days and the paid sickness allowance for each included patient. This is the basis for calculating the short- and long-term social costs of the incapacity to work due to MS.

If these secondary data are not available, or not available to a sufficient extent, the frequency of visits to specialists, inpatient facilities, and other medical service providers can be determined from the results of the patient questionnaire.

Additionally, the intervention’s implementation potential into the standard care system will be examined, because even if it can be demonstrated that patients benefit from the new form of care, this alone is no guarantee that it will be adopted as standard care. In that way the implementation potential is assessed through:

(14) Interviews and surveys with service providers (e.g. doctors, psychologists, nurses, medical assistants) on the acceptance of the intervention. This analysis aims to record supporting and hindering factors during the project period as a secondary outcome because the knowledge of these factors is a basic prerequisite for the subsequent implementation in regular care.
(15) Intervention costs: For the study period the individual interventions are remunerated to the service providers at flat rates. Based on time records and surveys, the actual costs of the individual interventions are determined to examine whether the compensatory allowances when transferred to standard care will ensure adequate future financing for the service providers even after the project duration.

The patients’ primary and secondary data are linked via a patient pseudonym. For this purpose, a trustee agency is involved.

Endpoints, outcomes, sources, and follow-ups are presented in detail in Table 2.

**Table 2:** Endpoints, outcomes, sources, and follow-ups

	Outcome	Measurement instrument or data source	Follow-ups
PRIMARY	Number of sick leave days	Routine data from the SHI funds Patient questionnaire and digital patient health record	-^*)^ Month 0, 6, 12, 18, 24 (and during quarterly consultations)
SECONDARY	Costs of the incapacity to work	Routine data from the SHI funds Human capital or friction costs approach based on primary data for sick leave days	-^*)^ Month 0, 6, 12, 18, 24 (and during quarterly consultations)
	Treatment costs/ frequency: • Inpatient care • Outpatient care • Rehabilitation • Medication • Remedies • Therapeutic appliances • Others	Routine data from the SHI funds Patient questionnaire	-^*)^ Month 0, 6, 12, 18, 24
	Out-of-pocket payments	Patient questionnaire	Month 0, 6, 12, 18, 24
	Implementation potential into standard care: • Acceptance • Intervention costs	Interviews and survey of service providers Process analysis, time records and surveys	Study month 24 & 36 Study month 12–36
	Reduction in earning capacity: •Subjective prognosis of gainful employment • Employment situation	Subjective prognosis of gainful employment scale (SPE) Patient questionnaire	Month 0, 12, 24 Month 0, 12, 24
	Physical status	Expanded Disability Status Scale (EDSS) Multiple Sclerosis Functional Composite (MSFC: T25-FW, 9-HPT)	Month 0, 6, 12, 18, 24
	Cognitive status Affective status Fatigue	Symbol Digit Modalities Test (SDMT) Depressions-Anxiety-Stress-Scale (DASS) Fatigue Scale for Motor and Cognitive Functions (FSMC)	Month 0, 6, 12, 18, 24
	Health-related quality of life	Multiple Sclerosis Quality of Life (MSQoL)-54 instrument EuroQol-questionnaire (EQ-5D-5L)	Month 0, 6, 12, 18, 24
	Patients’ adherence to therapy	Adherence Assessment Questionnaire (AAQ)	Month 0, 6, 12, 18, 24
	Self-Efficacy	Coping Self-Efficacy Scale (CSES)	Month 0, 6, 12, 18, 24
	Patient satisfaction with the new intervention	Client Satisfaction Questionnaire (ZUF-8)	Month 12, 24

*) Continuous recording within the claims data of the statutory health insurers

### Statistical analyses

The primary analysis will be performed as an intention-to-treat, ITT, analysis.

A regression model for count data with “zero inflation”, i.e. with a large proportion of cases without incapacity to work, is used to analyze the primary outcome. For this purpose, the zip command in the statistical package STATA is used. The statistical analyses of the other outcome variables are performed with generalized linear mixed-effects models [24].

These models are appropriate for the analysis of cross-sectional and longitudinal data on primary and secondary outcomes, which may be continuous (cost, quality of life) or categorical (early retirement) variables. Interdependencies between measurements in longitudinal data can be appropriately handled by including mixed effects. The application of the regression models to variables with different scale levels is made possible by the appropriate choice of the link function (e.g. identity for continuous variables, logistic for dichotomous variables) and the error distribution (e.g. normal distribution for continuous target variables, binomial distribution for dichotomous variables).

For data analysis with generalized linear mixed models, corresponding procedures are available in all relevant statistical packages (e.g. SPSS, SAS, Stata, R). Beyond these advantages of the chosen analysis model, it should be emphasized that with these models it is possible to specify and statistically test relevant hypotheses about the group, time, period, and interaction effects. These models also allow the handling of missing follow-up data.

For the analysis of cost data, special gamma regression models can be used according to the distributional properties of the dependent variable. With the help of suitable methods, the dispersion of costs and efficiencies is explained, i.e., those parameters are determined which have a significant influence on the costs and efficiencies of the measures.

Cost-benefit analysis: Based on the intervention costs and the saved costs (if applicable), the profitability of the measures is determined. In addition, the difference between intervention costs and the potentially saved costs for MS patients is compared to the effectiveness of the measures (cost-effectiveness analysis), to give information on the intervention’s advantageousness compared to other health policy instruments.

Institutional conditions: Based on the survey of the intervention costs and the acceptance of the intervention by the health care providers, the conditions for a successful implementation of the intervention as part of the standard care are elaborated and evaluated.

An interim analysis is not planned.

### Monitoring and data management

Before the start of recruitment, the neurological practices receive training on the interventions, use of the assessment instruments, the study documentation, the forwarding of the study documentation to the evaluating institutions, as well as the process and obligations regarding data validation. Online training is required, additionally, written and video-based training material is made available via the electronic health record platform and an access-restricted website.

Monitoring is carried out by the central project coordinator (BDN) and the evaluating institutions (UG and UMG). It starts directly after recruitment and the inclusion of the first patients. The principle of data Management has been described in a comprehensive data management plan.

## Discussion

To the best of our knowledge, this is the first study to consider multidisciplinary care in MS patients and its effects on health, gainful employment, and sick leave in Germany. In the project, patients with MS receive a bundle of interventions aiming to positively influence their ability to work and to participate in a self-determined life. The neurologist at the centre of the newly established network coordinates the care services offered by various service providers and, for the first time, works together with a preventive occupational physician. Tele-consultations and case discussions support this cooperation.

This complex intervention needs a highly integrated collaborative evaluation of health and health economic effects. In that way, primary and secondary data will be primarily matched, but due to the extensive collection of primary data within the project, a comprehensive evaluation of the new intervention is possible even in the absence of secondary data. The patient questionnaire and the documentation of MS-specific days of incapacity to work by the treating neurologists within the digital patient file make sufficient data available to analyze the primary endpoint, costs of incapacity to work and the course of treatment independently.

Complementing the patient perspective and the social and occupational participation outcomes achieved through the interventions, the perspective of service providers is also highlighted. Even though the new and comprehensive care for MS patients with the managing neurologist in the middle of the network can be understood as an innovation that goes beyond the current level of care. It is therefore of central importance that the new form of care is seen as useful and reasonable by the providers on the one hand, and that adequate financing can be ensured in the future on the other. The determination of the actual costs is thus indispensable for the transfer of this pilot implementation within the framework of MSnetWork.

Against this background, the study aims to improve the current employment situation of MS patients, considering disease-specific, therapeutic, psychosocial, and socioeconomic factors and the providers’ perspective to ensure such future treatment for MS patients.

If the project proves to be a success, a transfer for the treatment of other chronic diseases with an impact on the ability to work is conceivable and desirable.

## Data Availability

The study protocol is available as open access publication in agreement with SPIRIT [25] and CONSORT [26] criteria. The final trial dataset will primarily be available for the independent evaluation centres (UG and UMG) and the study coordinating centre (BDN). Data protection regulations restrict the use of the trial data for purposes and by institutions being agreed on by participants providing written informed consent. Any queries regarding data availability can be forwarded to the corresponding author. The trial results will be made available by scientific publication and reported to the funding body. Authorship eligibility guidelines for publishing peer-review journals will be applied.

## Abbreviations

9-HPT: 9-Hole Peg Test
AAQ: Adherence Assessment Questionnaire
BDN: Bundesverband Deutscher Neurologen (Professional Association of Neurologists)
CSE: Coping self-efficacy
CSES: Coping Self-Efficacy Scale
DASS: Depression, Anxiety and Stress Scale
EDSS: Expanded Disability Status Scale
EQ-5D-5L: EuroQol questionnaire, 5 dimensions, 5 level
FSMC: Fatigue Scale for Motor and Cognitive Functions
GWQ: Gesellschaft für Wirtschaftlichkeit und Qualität bei Krankenkassen (Society for Efficiency and Quality in Health Insurance Funds)
HRQoL: Health-related quality of life
ITT: Intention-to-treat
MS: Multiple sclerosis
MSFC: Multiple Sclerosis Functional Composite
MSQoL-54: Multiple Sclerosis Quality of Life-54 instrument
RCT: Randomized controlled trial
SDMT: Symbol Digit Modalities Test
SF-36: Short Form Health 36
SHI: Statutory Health Insurance
SPE-scale: Short scale measuring the subjective prognosis of gainful employment
T25-FW: Timed 25-Foot Walk
UG: University of Greifswald
UMG: University Medicine of Greifswald
VAS: Visual Analog Scale
ZUF-8: Patient Satisfaction Questionnaire
